# Diagnostic Workup of Primary Cutaneous B Cell Lymphomas: A Clinician's Approach

**DOI:** 10.3389/fonc.2020.00988

**Published:** 2020-06-23

**Authors:** Giulia Tadiotto Cicogna, Martina Ferranti, Mauro Alaibac

**Affiliations:** Unit of Dermatology, University of Padua, Padua, Italy

**Keywords:** primary cutaneous B-cell lymphomas, work-up, computerized tomography, positron emission tomography, bone marrow biopsy

Primary cutaneous lymphomas (PCLs) are a heterogeneous group of extranodal non-Hodgkin lymphomas, limited to the skin, with no evidence of extracutaneous disease at the time of diagnosis. PCLs are the most frequent extra-nodal lymphomas, with an incidence around 10 cases per million inhabitants per year. Among all PCLs, primary cutaneous B-cell lymphomas (PCBCLs) account for ~25–35%. According to the 2018 World Health Organization (WHO)-European Organization for Research and Treatment of Cancer (EORTC) classification for primary cutaneous lymphomas, PCBCLs are classified into three most common entities: primary cutaneous marginal zone B-cell lymphoma (PCMZL), primary cutaneous follicle-center lymphoma (PCFCL), and primary cutaneous diffuse large B-cell lymphoma-leg type (PCLCL-LT). PCMZL and PCFCL are considered clinically indolent and have an excellent prognosis, with 5-year survival rates higher than 90%. PCLCL-LT is less common and more aggressive, with 5-years survival rate lower than 60% (1).

PCBCLs must be distinguished from nodal or systemic malignant lymphomas involving the skin secondarily, which are characterized by different clinical behavior and prognosis, and require distinct therapeutic approaches. Thus, a correct staging workup is mandatory for an appropriate management.

The TNM classification system is the most widely used system for the assessment of the extent of non-lymphoid malignant diseases. The addition of item “B” (blood) to TNM was proposed to better stage patients with cutaneous T-cell lymphomas presenting as mycosis fungoides (MF) or Sézary syndrome (SS), which together account for the 75–85% of all PCLs. Nevertheless, TNM+B classification system is not applicable for non-MF/SS primary cutaneous lymphomas due to their different clinical behavior (2). For this purpose, in 2007 the International Society for Cutaneous Lymphomas (ISCL) and EORTC proposed new staging recommendations for cutaneous lymphomas other than MF and SS (3). According to these recommendations, an accurate staging should begin with a complete assessment of systemic symptoms and a physical examination. In every case blood tests should be performed, including a complete blood count with leukocyte differential, liver and kidney function analysis, serum levels of beta-2-microglobulin and lactate dehydrogenase (LDH). If laboratory tests are altered an immunophenotypic study of peripheral blood lymphocytes should be considered (3). Detection of chromosomal abnormalities in PCBCLs may be also useful for diagnosis, prognosis and therapy and it is generally performed using fluorescent *in situ* hybridization (FISH) (4). In particular, rearrangements of the MYC gene detected by FISH are present in a considerable percentage of patients with PCLCL-LT (32%) and patients exhibiting MYC rearrangement showed an inferior disease-specific survival and disease-free survival (5). Therefore, it may be useful to perform MYC-FISH in all newly diagnosed PCLBCL-LT. Furthermore, the mutational profile of the coding genome of PCLBCL-LT using whole genome sequencing may also help in the diagnosis in selected cases by the identification of a high mutational rate for MYD88, PIM1 and CD79B genes (6). The chromosomal translocation t(14;18)(q32;q21) is found in the majority of nodal follicular lymphomas and is responsible for overexpression of Bcl-2, a protein that prevents apoptosis of neoplastic cells. Immunohistochemical staining of PCFCLs generally showed low BCL2 expression intensity (about 69% of cases) or no expression (31%) whereas only 9% of PCFCL cases displayed the t(14;18) translocation using FISH analysis. However, the t(14;18) translocation with BCL-2 expression in PCFCLs was not associated with a worse prognosis and consequently identification of this translocation is not sufficient to predict clinical prognosis (7).

It has been suggested to perform in the indolent types of PCBCLs (PCMZL and PCFCL) the identification of *Borrelia* infection by antibody test using enzyme linked immunosorbent assay (ELISA) and by polymerase chain reaction (PCR) of *Borrelia* DNA in tumor tissue. To this regard, there is some evidence that *Borrelia* infection in North American and European endemic areas (from southern Scandinavia into the northern Mediterranean countries of Italy, Spain, and Greece, east from the British Isles into central Russia and the northeastern and north-central United States) may play a role in the pathogenesis of indolent type of PCBCLs because of chronic inflammation (8–11). This association may have therapeutic implications as antibiotic therapy (likewise to *Helicobacter pylori* infection in gastric MALT lymphoma) might be useful in treating these types of lymphomas. On the other hand, the largest available study aimed to evaluate the prevalence of *Borrelia* infection in patients with cutaneous lymphomas failed to demonstrate an association between PCBCL and this microorganism in non-endemic area (9). Consequently, diagnostic test for the identification of *Borrelia* infection have no clinical significance and should not be performed outside areas endemic for this microorganism. Conversely, in areas endemic for Borrelia infection, the identification of *Borrelia* infection by ELISA would constitute a rational choice in patients with these malignancies.

An appropriate imaging study should be performed in all patients with PCBCLs and includes either a chest, abdominal and pelvic computerized tomography (CT) scan with contrast or a whole body positron emission tomography (PET) technique together with CT scan, PET/CT scan (12, 13). In case of clinically detection of pathologically enlarged lymph nodes of the head and neck areas, the imaging should include the neck in order to evaluate the cervical lymph nodes. In the guidelines for the work up of PCBCLs it is not specified which imaging technique to choose; CT with contrast is a frequently used imaging method in clinical practice which can show the presence of enhanced lesions, however CT may not be able to diagnose inconspicuous skin lesions, non-enlarged lymph nodes containing PCL tumor cells and subclinical visceral metastasis (3, 13). Whole-body PET/CT can detect lesions through the difference in metabolic activity between malignant and normal cells, giving the possibility to detect ignored skin lesions and visceral involvement, even in the absence of an anatomic abnormality (14). Even though there is an increased evidence in using PET/CT for staging PCBCLs, it seems that the role of PET imaging, especially in detecting skin lesions, varies among lymphoma types. As a matter of fact, in a study conducted by Feuerman et al. in 2019, they demonstrate that PET/CT detected 100% of cases of cutaneous involvement in PCLCL-LT and only 11% of PCMZLs and 27% of PCFCLs. This could be due the difference in glucose metabolism among the various types of lymphoma; neoplastic cells of indolent lymphoma have usually a lower metabolic activity whereas neoplastic lymphocytes of aggressive subtypes have a higher glucose metabolism (15). In our opinion, a CT scan with contrast could be sufficient in indolent lymphomas (PCMZLs and PCFCLs), whereas in more aggressive types of malignancies, such as in PCLCL-LT, a PET/CT should be performed to achieve a better staging of the disease. If imaging shows the presence of lymph nodes that have a short axis larger than 1 cm, or have significantly increased PET activity, they should be sampled for tissue examination (3).

Next step in staging PCBCLs is the bone marrow biopsy, but the role of this diagnostic tool is controversial (16). According to the WHO-EORTC classification guidelines a bone marrow biopsy is indicated for PCLCL-LT because of the potential risk of extracutaneous spread, whereas it is optional in patients with PCMZL and PCFCL which have instead an indolent clinical behavior with a very low risk of dissemination (17). On the other hand, results of bone marrow biopsy examination in PCMZL or PCFCL with negative imaging study showed that in the PCMZL group 2% of patients showed bone marrow involvement whereas in the PCFCL group 11% had bone marrow involvement. Moreover, PCFCL patients with a positive bone marrow had a significantly worse prognosis when compared with patients without bone marrow involvement (5-year disease specific survival 63 vs. 95%). These results indicate that bone marrow investigation may be useful for staging patients with a PCFCL especially if it displays BCL-2 expression, whereas bone marrow examination appears to have limited value in patients with PCMZL (18, 19).

In summary, an appropriate diagnostic process has important implications for the management of patients with PCBCLs. The clinician starts with the diagnosis issued by the pathologist and may ask for additional molecular and/or microbiological investigations. The subsequent diagnostic work-up of PCBCLs should determine the extent of disease and can be achieved through a careful staging assessment which should be based on the biological behavior of the different subtype of PCBCLs, notably indolent or aggressive.

## Author Contributions

GT, MF, and MA have made substantial contributions to conception and design of the article and have all been involved in drafting the manuscript and revising it critically for important intellectual content. All authors contributed to the article and approved the submitted version.

## Conflict of Interest

The authors declare that the research was conducted in the absence of any commercial or financial relationships that could be construed as a potential conflict of interest.
